# Supplementary material for: A Proteomics Approach Identifies RREB1 as a Crucial Molecular Target of Imidazo–Pyrazole Treatment in SKMEL-28 Melanoma Cells
Source: Int J Mol Sci. 2024 Jun 20;25(12):6760. doi: 10.3390/ijms25126760 (PMC11203724; doi:10.3390/ijms25126760)
Supplement: Supplementary file 1 [file ijms-25-06760-s001.zip › Figure S2 Common and exclusive DEPs at 24h. 48h and 72h.pdf]

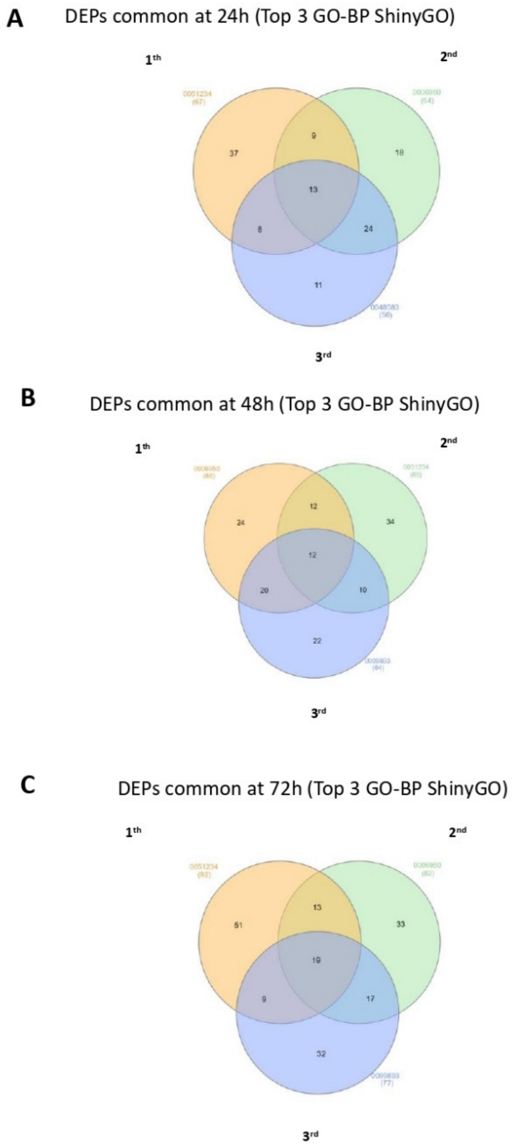

Figure S2. Common and exclusive DEPs at 24h. 48h and 72h.

Venn diagram shows the common and exclusive DEPs at three time points. Orange, green and blue colors represent respectively the first, second and third top ranked GO-BP on the base of the FDR values from which the Venn diagram originates. The lists of genes that we have used to generate this Venn diagram can be found in Table S2.
