# Supplementary material for: A Proteomics Approach Identifies RREB1 as a Crucial Molecular Target of Imidazo–Pyrazole Treatment in SKMEL-28 Melanoma Cells
Source: Int J Mol Sci. 2024 Jun 20;25(12):6760. doi: 10.3390/ijms25126760 (PMC11203724; doi:10.3390/ijms25126760)
Supplement: Supplementary file 1 [file ijms-25-06760-s001.zip › Figure S3 Enrichment of microRNA families modulating DEPs in 3e-treated Skmel-28 cells.pdf]

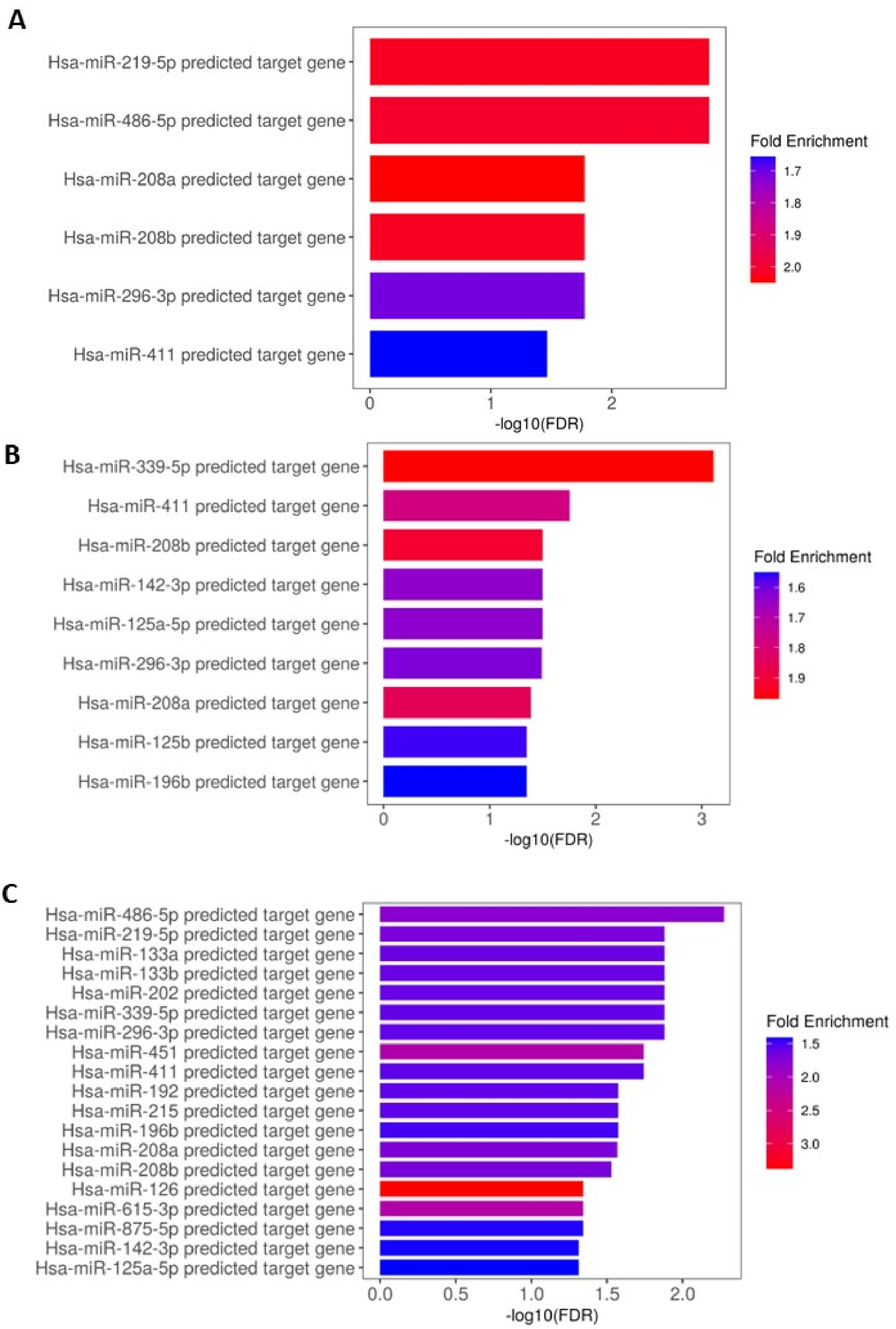

Figure S3. Enrichment of microRNA families modulating DEPs in **3e**-treated Skmel-28 cells.

Results from ShyniGO 0.80 microRNA target database are shown for analysis of microRNA putatively regulating the differential expressed proteins (DEPs) obtained from comparison between DMSO-treated and **3e**-treated cells for 24h (**A**), 48h (**B**) and 72h (**C**). In bar diagrams, fold enrichment is colour-scale represented (from the lowest blue to the highest red) and microRNA are ranked based on fold discovery rate (FRD) values (longer bar corresponds to lower FDR).
