# Supplementary material for: A Proteomics Approach Identifies RREB1 as a Crucial Molecular Target of Imidazo–Pyrazole Treatment in SKMEL-28 Melanoma Cells
Source: Int J Mol Sci. 2024 Jun 20;25(12):6760. doi: 10.3390/ijms25126760 (PMC11203724; doi:10.3390/ijms25126760)
Supplement: Supplementary file 1 [file ijms-25-06760-s001.zip › Figure S4 Prognostic value of deregulated transcription factors ZNF611, CUX1, TRAP2B, and RREB1.pdf]

A

B

C

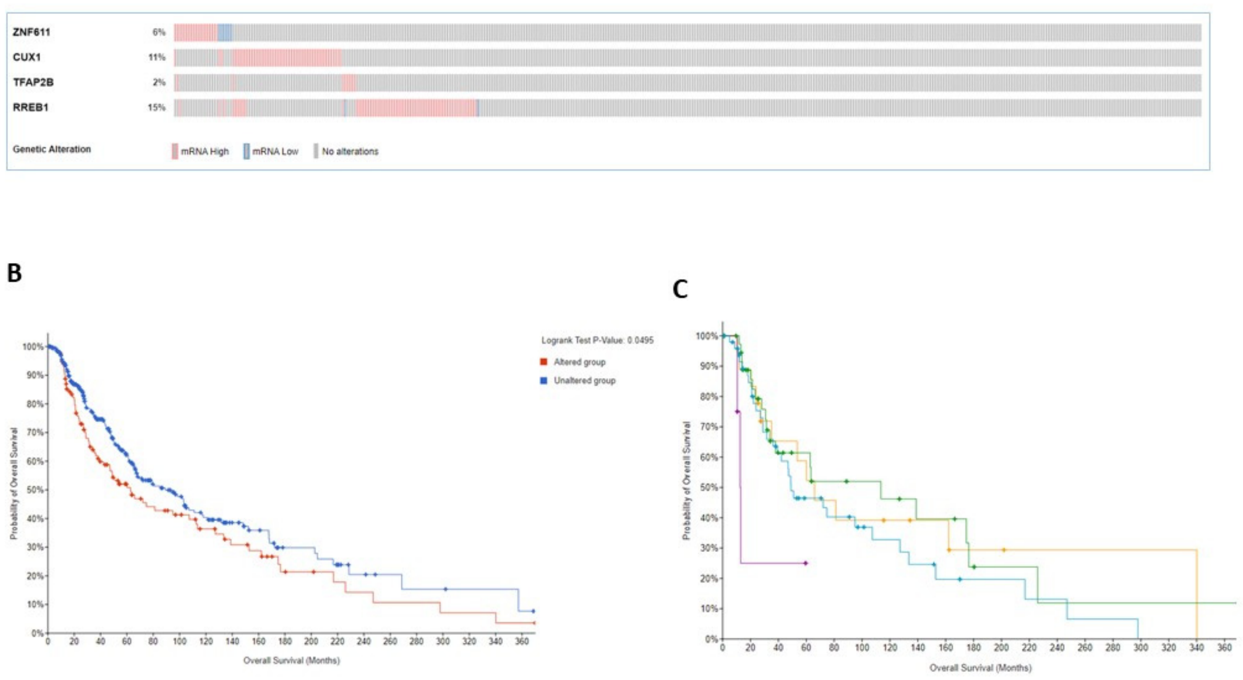

Figure S4. Prognostic value of deregulated transcription factors ZNF611, CUX1, TRAP2B, and RREB1.

(**A**) cBioPortal results concerning alteration in mRNA expression of the four deregulated TFs common to the three time-points following 3e treatment. The percentage of 441 patients carrying alteration in mRNA expression for one or more TFs is indicated on the left. Each bar indicates one patient, red and blue indicate up-regulated and down-regulated mRNA expression, respectively, while grey bars indicate patients without any mRNA alteration in these four genes. (**B**) Kaplan-Meyer curve showing the cumulative outcome in terms of probability of overall survival of all patients characterised by altered mRNA expression ZNF611, CUX1, TRAP2B, and RREB1 (red line) with respect with patients carrying no mRNA alterations (blue line). (**C**) Kaplan-Meyer curve showing the outcome in terms of probability of overall survival of patients grouped for alterations exclusively in ZNF611, CUX1, TRAP2B, or RREB1.
