# Supplementary figures and images for: A Proteomics Approach Identifies RREB1 as a Crucial Molecular Target of Imidazo–Pyrazole Treatment in SKMEL-28 Melanoma Cells
Source: Int J Mol Sci. 2024 Jun 20;25(12):6760. doi: 10.3390/ijms25126760 (PMC11203724; doi:10.3390/ijms25126760)

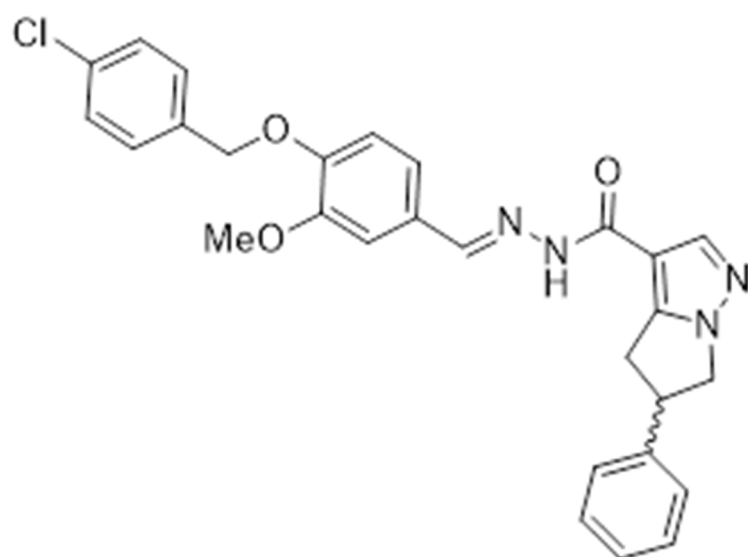

**Figure S1.** Chemical structure of compound **3e**

Supplement: Supplementary file 1 [file ijms-25-06760-s001.zip › Figure S1 Chemical structure of compound 3e.pdf]
